# Introducing the EpG^2^ System: Epigenomic Processes and the Emergent Genome

**DOI:** 10.3390/epigenomes9040049

**Published:** 2025-12-05

**Authors:** Edward A. Ruiz-Narváez

**Affiliations:** Department of Nutritional Sciences, University of Michigan School of Public Health, 2867 SPH I, 1415 Washington Heights, Ann Arbor, MI 48109, USA; eruiznar@umich.edu; Tel.: +1-734-647-0623

**Keywords:** epigenome, genome, chromatin, enhancer, risk allele

## Abstract

**Background/Objectives:** Current genomics research equates the genome with DNA sequence and treats the epigenome as a regulatory layer. This DNA-centric view obscures the fact that genomic identity arises through epigenomic processes. The objective of this article is to reinterpret published findings into a new theoretical framework: the EpG^2^ (Epigenome–Genome) system. **Methods:** This work develops a new conceptual framework by integrating published evidence from diverse domains—including enhancer biology, overlapping genomic functions, alternative coding frames, zygotic genome activation, and disease-associated loci—and reinterpreting these findings through the lens of epigenomic processes. **Results:** Evidence shows that enhancers emerge only through the interplay of sequence, transcription factors, and chromatin environment. At fertilization, paternal and maternal genomes remain separate, and a new genome emerges through coordinated epigenomic reprogramming or zygote genome emergence (ZGE). DNA sequence risk variants illustrate the concept of contextual risk alleles, whose effects shift across tissues and developmental stages as epigenomic contexts change. **Conclusions:** The EpG^2^ system reframes the genome as a processual, emergent entity generated and regulated by epigenomic processes, offering a paradigm for understanding genomic variation beyond DNA sequence.

## 1. Introduction

*“From now onwards space by itself and time by itself will recede completely to become mere shadows and only a type of union of the two will still stand independently on its own.”*

Hermann Minkowski, lecture at the 80th Meeting of German Natural Scientists, Cologne, 21 September 1908

In contemporary omics research, the genome and epigenome are usually treated as two distinct but related entities. The genome is often equated with the complete DNA sequence of an organism (U.S. National Cancer Institute, https://www.cancer.gov/publications/dictionaries/cancer-terms/def/genome accessed on 22 August 2025), while the epigenome is described as a collection of chemical compounds that regulate when and how genes are expressed (National Human Genome Research Institute, https://www.genome.gov/about-genomics/fact-sheets/Epigenomics-Fact-Sheet accessed on 22 August 2025). Popular metaphors reinforce this division: the epigenome is likened to an electrical switch that turns a gene on or off (see, for example, https://www.mpg.de/9910690/epigenetic-switch-obesity accessed on 22 August 2025), or to annotations on a musical score that guide performance [1]. In each case, the underlying assumption is that the genome enjoys ontological priority—genes must exist before they can be regulated, a light bulb must be present before it can be switched on, a score must be written before it can be annotated. By implication, one could imagine a genome without an epigenome, but not an epigenome without a genome.

This dichotomy, while intuitively appealing, is contradicted by growing evidence and obscures the emergent nature of the genome. DNA sequence alone does not determine the identity or activity of genomic elements. If the genome were nothing more than the DNA molecule, then sequence alone should suffice to specify the roles of protein-coding genes, regulatory sequences, and structural domains. Yet decades of functional genomics have demonstrated otherwise. Regulatory elements such as enhancers, silencers, and insulators do not pre-exist as fixed DNA stretches awaiting activation. Their functional identities emerge from the dynamic interplay of DNA sequence, interactions with DNA-binding proteins and chromatin-associated factors, chromatin modifications, and three-dimensional architecture [2,3]. Moreover, the same DNA locus can adopt distinct or even opposite roles across cellular or developmental contexts [4,5].

Taken together, these findings challenge the prevailing DNA-centric view. They suggest instead that the genome cannot be disentangled from the epigenome, and that both are inseparable components of a larger dynamic entity. In this work, I advance the theory of the EpG^2^ (EpiGenome–Genome) system, which conceptualizes the genome as an emergent entity resulting from epigenomic processes. Within this framework, the genome can be understood as the mapping of functional identities onto the DNA substrate—mappings that shift across time, cell type, and molecular environment. As a result, a single organism may harbor multiple genomes, even though its DNA sequence remains constant.

This reconceptualization has important consequences. It compels us to rethink how genomic elements are defined, how functional assays are interpreted, and how we search for the molecular basis of phenotypic variation and disease. More broadly, it points toward a necessary paradigm shift: just as relativity theory dissolved the separation between space and time into a unified space–time continuum, genomics must now move beyond the dualism of genome and epigenome toward the integrated EpG^2^ system.

In the sections that follow, I will illustrate this framework with concrete examples from current genomics and epigenomics research. These include the context-dependent identity of enhancers, the coexistence of multiple roles at a single DNA locus, and the emergence of distinct proteins from the same mRNA transcript. Together, they show that genomic function is not encoded in DNA sequence alone but arises from the dynamic, multilayered processes that constitute the EpG^2^ system.

## 2. Enhancers: From DNA Fragments to Emergent Entities

The first description of what we now call enhancers came from the classic study of Banerji, Rusconi, and Schaffner [6]. In this pioneering work, a rabbit β-globin gene was linked to fragments of SV40 viral DNA and transiently expressed in HeLa cells. The authors found a striking ~200-fold increase in transcription when SV40 DNA was present, localizing this effect to a 72 bp repeat element that could act in either orientation and over long distances. This discovery defined enhancers operationally as DNA fragments capable of boosting transcription, but because the assay measured RNA output without assessing chromatin or bound proteins, enhancers were initially conceptualized narrowly in terms of DNA sequence. Modern research has since shown that enhancers cannot be characterized by sequence alone. First, in vivo, enhancers are always bound by DNA-binding proteins and characterized by specific chromatin modifications (e.g., H3K27ac, H3K4me1) [3]. In fact, chromatin immunoprecipitation-sequencing (ChIP-seq) is one the most commonly used techniques to identify candidate enhancers [2]. Second, functional studies show that enhancer activity can persist despite extensive DNA sequence divergence, and conversely, DNA sequence conservation does not guarantee retained function. For example, heart enhancers in mice often lack strong sequence conservation yet remain active in vivo [7], and Drosophila enhancers display functional conservation through compensatory motif turnover despite high sequence divergence [8,9]. At the same time, many ultra-conserved or deeply constrained non-coding elements fail to act as enhancers at specific developmental stages [10,11].

One might object that enhancers already exist in the DNA molecule, lying latent until they are activated by the binding of transcription factors and chromatin modifications. In this view, the enhancer is present in the genome as a pre-existing element, and epigenomic processes merely switch it on or off. Although conceptually attractive, this perspective reduces enhancers to passive stretches of DNA, waiting to be activated. I argue instead that enhancers are emergent genomic entities, arising through the integration of DNA sequence, interactions with DNA-binding proteins, and chromatin context. As discussed above, neither enhancer identity (what an enhancer is) nor enhancer function (what an enhancer does) can be ascribed to sequence alone; both emerge from the dynamic interplay among sequence, proteins, and chromatin. The emergent enhancer thus possesses regulatory properties—such as distance-independent activation and combinatorial specificity—that none of its individual components exhibit in isolation.

Importantly, enhancers can also emerge de novo without any alteration of the underlying DNA sequence, simply through the overexpression of transcription factors. For example, Fu et al. [12] showed that upregulation of the pioneer factor FOXA1 in breast cancer cells produced thousands of new enhancers marked by H3K27ac that were absent under baseline conditions. Likewise, studies of super-enhancers show that high local concentrations of master transcription factors and cofactors can cluster to establish regulatory domains that reprogram cell identity [13,14]. These findings reinforce the thesis that enhancers are not latent DNA elements awaiting activation but emergent entities whose identity and function arise only from the integrated action of DNA sequence, transcription factors, and chromatin context.

## 3. One DNA Locus, Multiple Functional Identities

A corollary of the prevailing paradigm that equates the genome with the static DNA molecule is the assumption that a single DNA locus (i.e., a specific physical location in the DNA molecule) can possess only one functional identity. However, accumulating research shows that multiple functional identities can coexist within the same locus. Exonic enhancers (eExons) exemplify this principle: coding exons that simultaneously encode protein and function as transcriptional enhancers. For example, exons within *DYNC1I1* function as limb enhancers regulating the developmental genes *DLX5/6* [15,16]. Likewise, coding exons in *SORL1* and *PPARG* not only contribute to the protein sequence but also function as enhancers controlling the transcription of their own genes [17]. A further illustration is provided by loci that act as enhancers in one cellular context and silencers in another, a phenomenon observed in Drosophila and human cells, where transcription factor combinations and chromatin environment determine whether the same DNA element activates or represses transcription [4,5]. Kolovos and colleagues [18] proposed an integrated model that helps explain this duality: regulatory elements act by tethering promoters toward or away from transcriptional factories, so that the same locus can operate as enhancer, silencer, or even insulator depending on which transcriptional hubs and protein complexes it engages. A recent study in humans described a single element functioning simultaneously as an enhancer of *ATP2B4* and a silencer of *LAX1* in the same cellular context, underscoring that opposite regulatory functions can emerge from one DNA sequence [19]. Together, these cases demonstrate that functional identity is not encoded in the DNA sequence itself but emerges from its regulatory context.

## 4. When Functional Identity Emerges Outside the DNA

Functional identity in the genome is not restricted to the DNA molecule. Distinct functional identities can also emerge at the level of mRNA translation, where a single transcript may give rise to multiple, unrelated proteins. A well-known case is the *INK4a/ARF* locus, which encodes two tumor suppressors from overlapping reading frames: p16^INK4a^, acting through the retinoblastoma protein (pRB) pathway, and p14^ARF^ (p19^ARF^ in mouse), acting through the p53 pathway [20]. Here, exon 2 contributes to both proteins but is read in different frames, producing divergent functional identities from the same nucleotide sequence. Another case is the *GNAS* locus, where a single exon contains two overlapping +1 reading frames. These frames encode the G protein α-subunit variant XLαs and the structurally unrelated protein ALEX [21,22].

These cases show that functional multiplicity may arise not from changes in DNA sequence but from the translational machinery itself. A central mechanism is leaky ribosomal scanning, in which the scanning ribosomal 40S subunit bypasses an AUG codon in a weak Kozak context and initiates at a downstream start site. Leaky scanning, together with reinitiation, is a common occurrence in eukaryotic translation and provides a means to access alternative open reading frame (ORFs) [23,24]. In addition, genome-wide analyses confirm that upstream ORFs (uORFs) are pervasive and actively shape which coding sequences are translated, demonstrating that protein identity is highly sensitive to initiation context [25,26].

Protein mistranslation—the erroneous incorporation of amino acids during translation—constitutes another mechanism, alongside the use of alternative ORFs, through which distinct protein identities can emerge independently of changes in the DNA molecule. For example, in the fungal pathogen *Candida albicans*, the CUG codon is ambiguously decoded (predominantly as serine, but sometimes leucine), leading to proteome-wide variation in amino acid identity at many positions and reshaping functional properties of hundreds of proteins [27,28]. In addition, protein mistranslation can have functional implications that extend beyond the misincorporated proteins themselves. In *Candida albicans*, adaptive mistranslation of the CUG codon does not merely diversify the proteome but also remodels cellular physiology and genome architecture. Hypermistranslating strains evolve fluconazole resistance more rapidly than wild-type strains and exhibit widespread genomic alterations, including loss-of-heterozygosity events, aneuploidy, and large-scale changes in gene expression [29]. Thus, mistranslation exemplifies how functional information can emerge and propagate across molecular levels, linking transient decoding variability to long-term genomic reorganization.

Beyond the layers of functional information that arise through alternative ORF usage and translational decoding, RNA modifications now appear as a further domain in which identity and regulatory capacity transcend the static DNA template. Chemical marks on RNA—such as N^6^-methyladenosine (m^6^A), 5-methylcytosine (m^5^C), and pseudouridine (Ψ)—act as dynamic regulators of gene expression, influencing transcription, translation, RNA structure, stability, and decay [30,31]. More strikingly, recent evidence shows that the machinery that writes, erases or reads these marks is influenced by, and in turn influences, chromatin accessibility and transcriptional dynamics [32,33]. These observations place RNA modifications within the recursive loop that defines the emergent genome: DNA gives rise to RNA, which is chemically modified, which modulates translation and feeds back to chromatin and transcriptional programs. In the EpG^2^ framework, the epitranscriptome represents an intermediate emergent subsystem, not a passive carrier of sequence information but an active modulator of system identity, capacity, and behavior.

Taken together, these examples highlight that genomic identity can emerge independently of the DNA sequence. Through alternative frame usage, context-dependent initiation, and mistranslation, a single transcript can give origin to distinct functional proteins.

## 5. The EpG^2^ System

**Figure 1:**
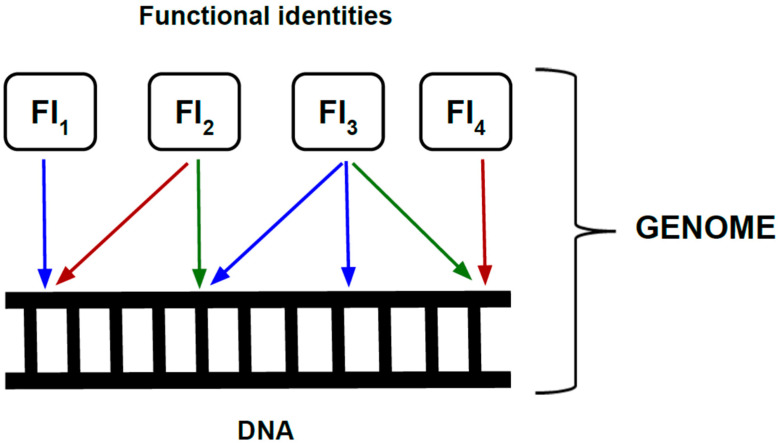
The genome as an emergent entity. In the EpG^2^ framework, the genome is defined as the set of functional identities (FIs) that map onto DNA through epigenomic processes. A single DNA locus can take on different functional identities—such as enhancer, promoter, coding region, or silencer—depending on biological context. Colored arrows illustrate that these mappings are context-dependent, varying across cell types, developmental stages, and environmental states. Through this dynamic mapping, DNA becomes instantiated into the emergent genome, whose composition changes with context.

First, I propose an updated definition of the genome. The genome is the set of DNA elements that acquire functional identity through epigenomic processes (Figure 1). A DNA sequence becomes part of the genome only when it is assigned a functional role—such as gene, enhancer, silencer, promoter, or insulator—within a specific cellular and developmental context. Because these mappings of function onto sequence vary across time and cell type, the same organism can harbor multiple genomes despite sharing a constant DNA sequence.

How then should we define the epigenome? Current definitions emphasize chemical changes to DNA (e.g., cytosine methylation) and histone modifications that regulate gene expression. I propose a broader definition: the epigenome is not only what tells the genome what to do but what defines what the genome is. In this sense, the epigenome is the set of processes that drive the emergence of a particular genome in a given cell type and developmental stage. This definition can be extended to include mechanisms that regulate the activity of the emergent genome, making the epigenome a multi-level system: it both establishes genomic identity and modulates genomic function.

**Figure 2:**
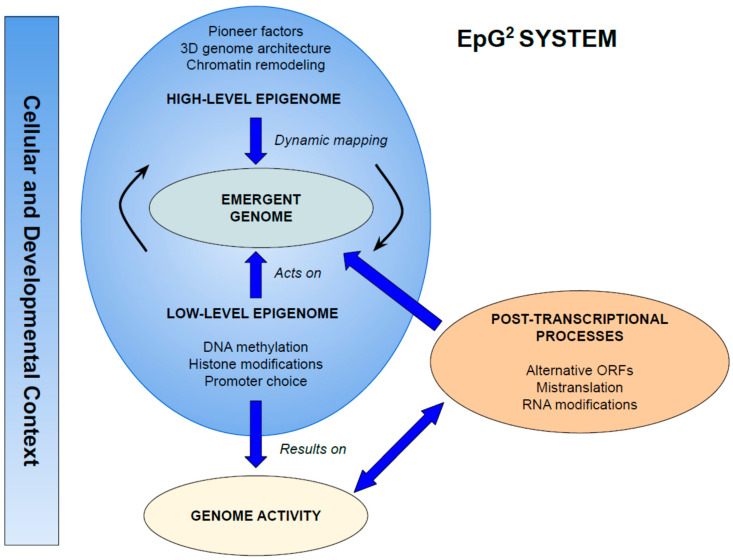
The EpG^2^ system: the multi-level epigenome and the emergent genome. The EpG^2^ system depicts the genome as an emergent entity generated by hierarchical yet interdependent epigenomic processes. The high-level epigenome (e.g., pioneer factors, chromatin remodeling, 3D architecture) gives rise to the emergent genome, whose curved arrows indicate continuous, context-dependent remapping. The low-level epigenome (e.g., DNA methylation, histone modifications, promoter choice) bridges the emergent genome and genome activity, modulating its function. Post-transcriptional processes such as alternative ORF use, protein mistranslation, and RNA modifications originate from genome activity but also feedback to reshape the emergent genome. All interactions occur within a cellular and developmental context, underscoring the genome’s dynamic, processual nature.

The EpG^2^ (EpiGenome–Genome) system captures this dual role as the integrated biological network through which the genome emerges from epigenomic processes (Figure 2). It operates at two complementary levels:

1. High-level epigenome:mechanisms that establish the identity of genomic elements by mapping functional roles onto DNA, thereby generating the emergent genome itself.
2. Low-level epigenome:mechanisms that regulate the activity of the emergent genome, including transcriptional output, alternative promoter usage, and chromatin dynamics.

In this view, the genome is not equivalent to the DNA sequence. Rather, it is the set of DNA elements endowed with functional identity by high-level epigenomic processes and dynamically regulated by low-level mechanisms. This two-level organization explains how a single organism, with a stable DNA sequence, can generate multiple genomes across developmental stages and cell types. The key concepts introduced in this work are summarized in Table 1.

## 6. Discussion and Implications

### 6.1. Elucidation of Mechanisms Leading to Genome Emergence

The present work claims that the genome is not equivalent to the DNA sequence, but an emergent entity that comes into being through multi-level epigenomic processes within the EpG^2^ system. The idea that the genome is more than the DNA molecule has been proposed by other researchers, for example, Heng and colleagues, who developed the concept of genome chaos to describe rapid and massive genome reorganization under stress conditions [34,35]. In their view, cancer evolution often proceeds through episodes of genome chaos in which chromosomal restructuring generates new systems of information and enables punctuated evolutionary transitions. From the perspective of the EpG^2^ system, genome chaos can be understood as an extreme instance of high-level epigenomic processes: moments when the mapping of functional identities onto DNA is destabilized and reorganized, giving rise to emergent genomic configurations with altered regulatory landscapes. Clinically, such chaotic remapping provides a plausible explanation for the capacity of tumors to evolve drug resistance and acquire aggressive phenotypes [35].

At the same time, the EpG^2^ framework extends beyond pathological contexts to encompass normal developmental and physiological conditions. A paradigmatic case is what is conventionally termed zygotic genome activation (ZGA). However, I argue that this process is better described as zygotic genome emergence (ZGE). The term activation implies that the zygotic genome already exists in a latent form, merely awaiting a switch to be flipped. In contrast, I propose that fertilization does not activate a pre-formed genome but initiates the formation of a new genomic entity—one that emerges through a dynamic process rather than simply being switched on. Multiple lines of evidence demonstrate that, at the moment of fertilization, there is no unified zygotic genome; instead, the maternal and paternal genomes remain distinct. The zygotic genome subsequently emerges through epigenomic processes that reconfigure these separate genomes into a new, integrated entity. First, in mammals, following fertilization, the maternal and paternal genomes remain physically segregated within distinct pronuclei. This segregation is maintained by the formation of two separate microtubule spindles that align with each other but preserve the spatial separation of both genomes until the first mitotic division [36]. Notably, the parental genomes continue to occupy distinct nuclear compartments in the two- and four-cell embryo [37,38]. Second, the timing of epigenetic reprogramming differs between the paternal and maternal genomes. Both undergo profound yet asymmetric epigenetic remodeling after fertilization: the paternal genome experiences rapid, active DNA demethylation, whereas the maternal genome is largely protected from this process and demethylates more gradually [39]. Third, the establishment of 3D chromatin architecture in the zygote is a dynamic and asymmetric process, characterized by distinct timing and structural features between the maternal and paternal genomes. After fertilization, the paternal genome undergoes rapid protamine-to-histone exchange and decondensation, quickly acquiring loops, weak TADs, and clear A/B compartmentalization that partially reflect its inherited 3D organization from sperm chromatin [40,41]. In contrast, the maternal genome enters the zygote in a mitotic-like condensed state and must rebuild its higher-order structure de novo [40,41]. These initial asymmetries in chromatin organization gradually diminish as the zygotic cell cycle progresses, reaching near-complete convergence by the 8-cell stage [42]. Finally, in humans, transcriptional activation proceeds asymmetrically, beginning from the paternal genome. Transcriptomic analyses of androgenetic and parthenogenetic embryos reveal that transcription is first initiated from the paternal genome at the 8-cell stage, while the maternal genome remains largely inactive until the morula stage [43]. Thus, at fertilization there is not one genome but two distinct genomic systems whose gradual coordination through epigenomic processes gives rise to a new genomic entity: the emergent zygotic genome.

Recent studies have proposed a useful division of regulatory factors involved in early development into two classes: licensors, which establish a permissive nuclear environment and control the timing of competence, and specifiers, transcription factors that activate particular sets of zygotic genes [39]. In the framework of the EpG^2^ system, I interpret this division as reflecting two levels of epigenomic processes: high-level mechanisms that configure nuclear organization and global accessibility, and lower-level mechanisms that specify gene-by-gene regulatory outcomes. Pioneer factors (PFs) exemplify the interdependence of the two levels of epigenomic processes. By binding nucleosome-embedded motifs and opening compact chromatin, PFs perform a licensor-like function: they establish global accessibility and contribute to the nuclear environment required for transcriptional competence [40,44]. At the same time, many PFs directly specify developmental programs—the zinc-finger protein Zelda primes enhancers for patterned gene expression in Drosophila, while Dux initiates the 2-cell transcriptional program in mammals [39]—thus functioning as specifiers as well. This duality of PFs demonstrates how high-level and low-level processes are entangled in practice. The capacity of PFs to operate simultaneously at different levels shows that genome emergence is produced by the integration of nuclear reorganization and gene-specific regulation.

Whereas Heng’s theory emphasizes genome reorganization under crisis [34,35], the EpG^2^ system underscores that genome emergence is an inherent, ongoing process. ZGE makes this principle visible: a new genomic entity arises from initially separate maternal and paternal genomes, assembled by high-level epigenomic processes. The EpG^2^ system proposes that similar dynamics continue throughout development, differentiation, and reprogramming, as epigenomic processes re-map functional genomic identities into DNA. Integrating these perspectives suggests a spectrum: from the steady-state emergence of the genome in ZGE and normal physiology to the disruptive remapping of genome chaos under stress and disease.

### 6.2. Genomic Variation and Disease

An active field of research is the elucidation of the hereditary basis of disease. In practice, this has meant asking how DNA sequence variation affects disease risk and severity. However, as proposed in the present work, the genome is not equivalent to the DNA sequence but an emergent entity that varies across cell types and developmental times. Consequently, the same physical positions along the DNA molecule may carry different functional identities depending on cellular context. Standard approaches such as genome-wide association studies (GWAS) and whole-genome sequencing therefore face an intrinsic limitation: they measure variation in the DNA sequence, but this is not necessarily the same as genomic variation.

The case of exonic enhancers (eExons) makes this point clear. Coding exons can simultaneously function as enhancers, regulating not only the gene in which they reside but sometimes distant genes. Mutations in eExons may alter enhancer activity and cause disease, even if the protein sequence remains unchanged [45], and dual-use exons play cell-specific regulatory roles [17]. These findings highlight the coexistence of multiple functional identities within the same DNA sequence—protein-coding and enhancer—precisely the type of genomic variation that the EpG^2^ system predicts.

A related limitation emerges when linking GWAS hits to gene regulation. Multiple studies report that only a minority of GWAS associations clearly colocalize with steady-state eQTLs, raising the problem of missing regulation. For autoimmune traits, joint likelihood colocalization across three major immune cell types identified shared genetic effects in only ~25% of loci, implying that most risk mechanisms are not captured by basal expression changes in those cells [46]. Consistently, a cross-trait analysis found limited evidence that baseline expression of trait-related genes explains nearby GWAS signals using colocalization, transcription-wide association, or annotation-based approaches [47]. In a targeted study of validated hypercholesterolemia genes and LDL-C GWAS loci, colocalization in relevant tissues was observed for only ~26% of loci [48]. Finally, aggregated across 42 traits, only ~11% of heritability was mediated by bulk-tissue eQTLs from the Genotype-Tissue Expression (GTEx) project, suggesting that standard eQTL resources capture only a limited portion of the regulatory architecture underlying complex disease [49].

From the perspective of the EpG^2^ system, these observations are expected: mapping functional identities onto DNA is context dependent. eQTL effects can change with cell type, activation state, developmental time, and in vivo disease environment. Recent single-cell analyses show that roughly a third of cis-eQTLs in memory T cells are cell-state dependent, with autoimmune risk variants enriched in such state-dependent eQTLs—precisely the kind of context sensitivity that bulk assays obscure [50]. In vivo immune-cell atlases further reveal broad cell type- and disease context-specificity of eQTLs across 28 immune cell subsets from patients with immune-mediated diseases, highlighting the dynamic and context-dependent effects of eQTLs [51]. Even in population whole-blood cohorts, thousands of context-dependent eQTLs emerge when accounting for cell composition and interferon signaling, or transcription factor modulated effects [52].

**Figure 3:**
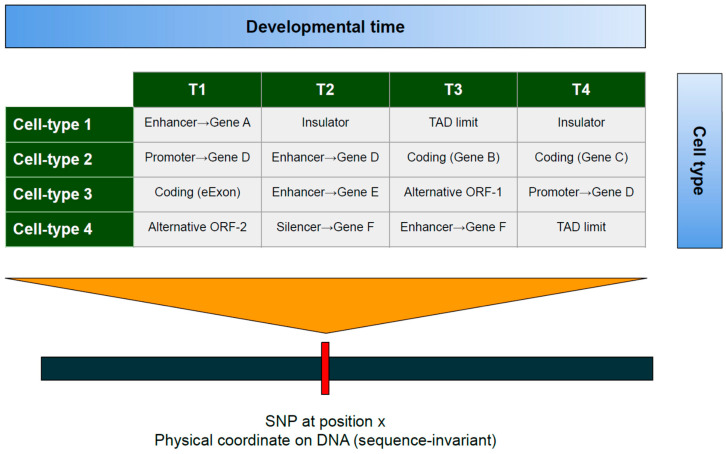
The contextual risk allele: cell-type- and time-dependent functional identities. The figure depicts a contextual risk allele, a single-nucleotide polymorphism (SNP) whose function changes across cell types (rows) and developmental times (columns T1–T4). Although its DNA position is constant, the same locus acquires different roles (e.g., enhancer, promoter, silencer, insulator, or coding sequence) depending on the genomic mapping. This variability shows that genetic risk is not an intrinsic property of DNA sequence but a consequence of context-dependent mappings within the EpG^2^ system, where functional identity emerges from dynamic interactions between the genome and multi-level epigenomic processes.

The EpG^2^-system theory reframes these findings: missing regulation reflects missing context. Because functional identities vary across cell types and developmental times, the same variant can participate in multiple genomic identities. What appears as a failure to colocalize in bulk, steady-state assays may in fact be a context-restricted regulatory effect that only manifests in specific cell states, developmental windows, or disease milieus. Under the EpG^2^-system theory, the search for causal SNPs should thus prioritize context-resolved regulatory maps (cell-type, state, time), single-cell and perturbational eQTL designs. Within this framework, I propose the concept of the contextual risk allele: a variant whose contribution to disease depends not only on its DNA sequence but also on the epigenomic processes that embed it within a particular emergent genomic identity (Figure 3).

### 6.3. The EpG^2^ System Within the Evolutionary Development Biology Framework

At its core, the EpG^2^ theory reframes the genome as a process—specifically, as the mapping of functional identities onto DNA—rather than as a fixed thing. It is an emergent system arising from epigenomic processes. This theoretical orientation of the EpG^2^ system aligns with the principles of evolutionary developmental biology (Evo-Devo). Evo-Devo emphasizes that evolutionary change depends not only on alterations in DNA sequence but also on the dynamics of developmental processes that interpret internal and external cues [53,54]. In this sense, development serves as the bridge between genotype and phenotype. The EpG^2^ system extends this logic to the molecular level, proposing that the genome itself is a developmental product—an entity that emerges through epigenomic processes.

This conceptual alignment between the EpG^2^ and Evo-Devo frameworks opens the way to reinterpret evolutionary mechanisms through the lens of genomic emergence. A paradigmatic example is genetic assimilation, the process by which a phenotype initially induced by environmental conditions becomes genetically stabilized through selection [55]. In his classic experiments on Drosophila, Waddington demonstrated that exposing embryos to heat shock produced an altered wing phenotype that, after several generations of selection, appeared even in the absence of stress [55]. The interpretation was that environmental perturbation revealed latent developmental potential, which genetic variation then fixed.

A non-exhaustive set of mechanisms proposed for genetic assimilation includes: (1) selection acting on pre-existing genetic variation [56]; (2) cooperative interactions between genetic and epigenetic factors, whereby natural selection incrementally replaces epigenetically induced phenotypes with genetic mutations over generations [57]; (3) disruption of canalization processes—such as through alterations in molecular chaperones like Hsp90—that reveal cryptic genetic variation or activate transposable elements, generating heritable mutations [58]; and (4) selection on stress-induced de novo mutations that stabilize previously plastic phenotypes [58]. From the perspective of the EpG^2^ system, genetic assimilation can be reinterpreted as the stabilization of an emergent genomic configuration. The initial environmental perturbation acts on high-level epigenomic processes creating a new mapping of functional identities onto DNA. Repeated induction of this state increases the probability that mutations or stable epigenomic modifications will reinforce the new genomic configuration. Over time, the new emergent genomic mapping would be selected and become part of the developmental process.

Because genomic mappings are time- and cell-dependent, the EpG^2^ system offers new ways of understanding developmental plasticity—the ability of developing organisms to adjust their trajectories in response to internal or external cues [59]— which is often balanced by canalization, the tendency of development to produce stable outcomes despite perturbation [60]. Within the EpG^2^ framework, this balance reflects the interaction between high-level epigenomic processes that establish and stabilize genomic identity, and low-level epigenomic mechanisms that regulate its activity and responsiveness. Together, these two layers allow the genome to remain both robust and adaptable, preserving identity while remaining open to developmental and evolutionary innovation.

This dynamic can be illustrated at the molecular level. Because functional identities emerge through time-specific epigenomic mappings, certain genomic elements may acquire regulatory roles only during restricted developmental windows. For instance, enhancers and exonic enhancers (eExons) can transiently assume enhancer activity at critical stages of differentiation or morphogenesis, responding to internal signaling gradients or external environmental cues. Once these temporal mappings fulfill their regulatory roles, they may disappear or revert to alternative functional states without any change in the underlying DNA sequence. Such temporally emergent functional identities illustrate that the genome functions as a process rather than a fixed structure, one that is continually rewritten by epigenomic dynamics to respond to developmental and environmental demands.

## 7. Summary

The EpG^2^-system theory advances a shift from a DNA-centric paradigm to one in which the genome is understood as an emergent entity shaped by epigenomic processes. This framework introduces key conceptual innovations, including the reframing of zygotic genome activation as zygotic genome emergence (ZGE) and the definition of contextual risk alleles, variants whose effects depend on the epigenomic identities in which they are embedded. By highlighting that genomic variation is not equivalent to DNA sequence variation, the EpG^2^ system explains phenomena such as enhancer plasticity, overlapping functional roles of DNA loci, and the problem of missing regulation in GWAS results. The implications are broad: functional genomics, disease mapping, and translational research must move beyond static sequence-based approaches to embrace the dynamic, context-dependent mappings that constitute the emergent genome. In this sense, the EpG^2^ system offers not only a new theoretical framework but also a roadmap for future experimental and clinical genomics.

## Figures and Tables

**Table 1:** Key concepts introduced in the EpG^2^ system framework.

Concept	Definition
Genome	More than DNA sequence. The genome is the set of DNA elements that acquire functional identity through epigenomic processes. It is an emergent entity, varying across cell types and developmental stages.
Epigenome	The set of processes that establish and regulate genomic identities. The epigenome both defines what the genome is and modulates how it functions.
Functional identity	The functional role a DNA element acquires in a given context—such as enhancer, promoter, silencer, insulator, or coding region—emerging from interactions among sequence, DNA-binding proteins, and chromatin environment and architecture.
EpG^2^ system	The integrated Epigenome–Genome system. High-level epigenomic processes map functional identities onto DNA, producing the emergent genome; low-level processes regulate its activity.
Zygote genome emergence (ZGE)	Conventionally termed zygotic genome activation. Reframed as the coming-into-being of a new genome through epigenomic reprogramming, as maternal and paternal genomes—initially separate—are integrated into a totipotent system.
Contextual risk allele	A genetic variant whose effect on disease is context dependent. Its functional identity changes across cell types, developmental stages, or environmental states, reflecting the epigenomic processes that embed it in different emergent genomic functional identities.

## Data Availability

No new data were created or analyzed in this study. Data sharing is not applicable to this article.
